# Testosterone levels positively linked to muscle mass but not strength in adult males aged 20–59 years: a cross-sectional study

**DOI:** 10.3389/fphys.2025.1512268

**Published:** 2025-04-15

**Authors:** Wei Zhang, Zhaohui Cui, Dayong Shen, Li Gao, Qingyun Li

**Affiliations:** ^1^ Department of Neurology, The Affiliated Hospital of Xuzhou Medical University, Xuzhou, China; ^2^ Department of Orthopedic Surgery, Heze Municipal Hospital, Heze, China; ^3^ Department of Endocrinology, The Affiliated Hospital of Xuzhou Medical University, Xuzhou, China

**Keywords:** sex differences, testosterone, muscle mass, muscle strength, NHANES

## Abstract

**Background:**

The relationship between testosterone levels and muscle mass and strength remains contentious. This study aimed to explore the relationship among total serum testosterone levels, muscle mass, and strength in young to middle-aged adults.

**Methods:**

The analysis included 4,495 participants (age 39.2 ± 0.2 years, mean ± SE) from the National Health and Nutrition Examination Survey conducted between 2011 and 2014. Weighted regression models were used to assess the association of testosterone levels with muscle mass and strength.

**Results:**

For male participants, log_2_-transformed testosterone levels were positively associated with appendicular lean mass adjusted for body mass index (β: 0.05, 95% confidence interval [CI]: 0.03–0.07, *P* < 0.001) and negatively associated with low muscle mass (odds ratio: 0.40, 95% CI: 0.24–0.67, *P* = 0.006). However, no significant association was found between testosterone levels and grip strength (β: 1.16, 95% CI: 0.26 to 2.58, *P* = 0.086) or low muscle strength (odds ratio: 0.51, 95% CI: 0.25–1.04, *P* = 0.059). For female participants, no significant association was observed between testosterone levels and muscle mass (β: 0.01, 95% CI: 0.02 to −0.01, *P* = 0.294) or muscle strength (β: 0.14, 95% CI: 0.45 to 0.73, *P* = 0.508). Restricted cubic spline analysis revealed a linear relationship between total testosterone levels and appendicular lean mass adjusted for body mass index in male participants (nonlinear: *P* = 0.367).

**Conclusion:**

Our study indicates that testosterone levels are positively associated with muscle mass but not with muscle strength in young to middle-aged males.

## 1 Introduction

As individuals age, their body composition undergoes significant changes, including a decrease in skeletal muscle mass and strength (Dodds et al., 2016). This reduction is closely linked to a decline in muscle function, which increases the risk of adverse outcomes, such as frailty, falls, fractures, physical disability, and loss of independence (Cruz-Jentoft et al., 2019). With the increase in the global elderly population, the decline in muscle mass and strength has emerged as a critical issue with substantial implications for both the economy and public health. Several factors contribute to this decline, including malnutrition, immobility, neurological changes, and chronic illness along with hormonal changes related to aging, such as alterations in insulin-like growth factor-1 (IGF-1), growth hormones, sex hormones, and glucocorticoids (Coletta and Phillips, 2023; Priego et al., 2021).

Testosterone, the primary androgen hormone, is predominantly produced in the testicles, in smaller quantities, in the ovaries. It is essential for the development and maintenance of male reproductive tissues, sexual function, muscle and bone health, and overall wellbeing in both men and women. Additionally, it plays a significant role in maintaining muscle health. Research has demonstrated that testosterone activates satellite cells, promoting myonuclear accretion and replenishing the satellite cell pool (Kadi, 2008). It also influences pluripotent stem cells, favoring their commitment to myogenic lineage while inhibiting adipogenic differentiation (Bhasin et al., 2003). However, the evidence regarding the association between low testosterone levels and muscle mass and strength is inconsistent (Nam et al., 2018). For example, a cross-sectional study reported a significant positive relationship between total testosterone and muscle strength (Schaap et al., 2005), whereas a longitudinal analysis of two independent samples of older men found no association between low testosterone levels and low muscle strength (Schaap et al., 2008). While testosterone’s role in muscle health is well-established in older men, its impact in young to middle-aged adults and women remains understudied. This gap is critical for understanding early interventions and sex-specific hormonal contributions to muscle maintenance.

National Health and Nutrition Examination Survey (NHANES), a major epidemiological survey conducted by the National Center for Health Statistics (NCHS), provides a large, nationally representative sample of the US population (Zipf et al., 2013). It employs standardized and validated methods to measure testosterone levels and muscle mass and strength. In this study, we focused on the young to middle-aged population, which is an understudied demographic in preventive healthcare and chronic disease management. Additionally, the comprehensive set of covariates that we adjusted for, providing a more nuanced understanding of the relationship between testosterone, muscle mass, and strength in this demographic.

## 2 Methods

### 2.1 Data sources

The NHANES consists of voluntary US-based surveys administered every 2 years to assess the health of non-institutionalized individuals through interviews, physical examinations, and diagnostic evaluations (Zipf et al., 2013). The NHANES gathers comprehensive information on various health topics, including demographics, socioeconomics, diet, and health-related issues through in-home interviews, followed by blood sampling at mobile examination centers. The study protocol for the NHANES was reviewed and approved by the NCHS Research Ethics Review Board, and all participants provided written informed consent upon enrollment. This study follows the Strengthening the Reporting of Observational Studies in Epidemiology (STROBE) guidelines (von Elm et al., 2007).

### 2.2 Study design and population

**FIGURE 1:**
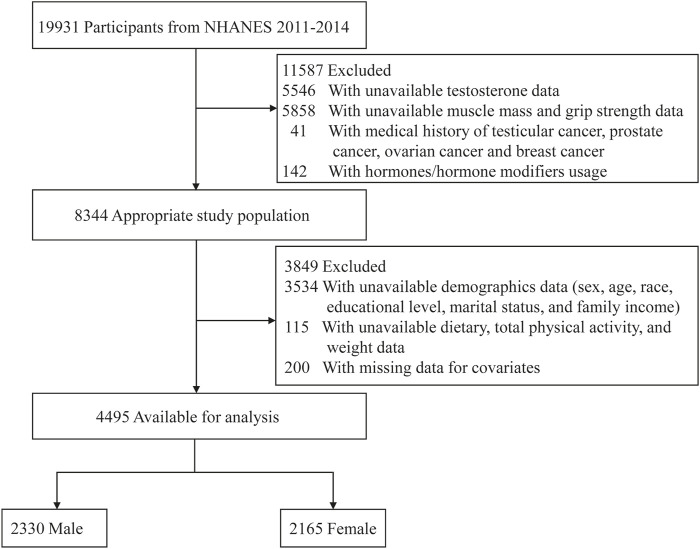
Flow diagram of the screening and enrollment of study participants.

This study analyzed data from two NHANES survey cycles (2011–2014), which were selected because they included data on testosterone (2011–2016) as well as the muscle mass (2011–2018) and muscle strength (2011–2014). Exclusion criteria included participants with unavailable data on testosterone (5,546) or muscle mass and strength (5,858); a medical history of testicular, prostate, ovarian, or breast cancer (Park et al., 2020); treatment with hormones or hormone modifiers (142); missing demographic data (3,534); missing information on diet, physical activity, and weight (115); and other covariates (200). Figure 1 shows a schematic representation of the participant enrollment procedure.

### 2.3 Measurement of testosterone

Serum blood samples were collected from participants aged ≥6 years during NHANES clinic visits. Total serum testosterone levels were measured using isotope dilution liquid chromatography-tandem mass spectrometry (ID-LC-MS/MS) method. The detection limits were constant for all of the analytes in the NHANES dataset. Specific subgroups (e.g., adolescents, older adults) are generally lower and may fall below the lower limit of detection. Consequently, this can lead to the exclusion of a significant number of minors from analyses involving testosterone levels. In addition, some participants did not undergo blood tests, this could be due to participant refusal, scheduling conflicts, or other logistical issues.

### 2.4 Assessment of appendicular lean mass (ALM) and handgrip strength

Dual-energy X-ray absorptiometry (DXA) scans using a Hologic QDR-4500A fan-beam densitometer were conducted to assess bone-free lean mass. Certain participants, such as pregnant individuals or those with contraindications to DXA, and self-reported weight over 450 pounds or height over 6′5”(DXA table limitation) were excluded. ALM was defined as the sum of the lean masses of both arms and legs, expressed in kilograms. To ensure accurate and reliable ALM calculations, participants who did not undergo the full set of DXA scans for both arms and legs were excluded. ALM adjusted for body mass index (BMI) was calculated, and low muscle mass was defined according to the Foundation for the National Institutes of Health (FNIH) criteria (ALM_BMI_: men, <0.789; women, <0.512) (Studenski et al., 2014). Handgrip strength, assessed using a dynamometer, served as a surrogate measure of muscle strength. Participants were excluded if they were unable to hold the dynamometer with both hands, and individuals who had undergone surgery on either hand within the past 3 months were not tested on the affected hand. The highest grip strength value (GS_MAX_) for each hand was included in the analysis, with low muscle strength defined as < 26 kg for men and <16 kg for women according to the FNIH sarcopenia project guidelines (Studenski et al., 2014).

### 2.5 Covariates

Based on the literature, demographic factors (age, gender, education, race, and income) directly influence adherence to exercise regimens and health behaviors, thereby affecting muscle health outcomes (Vezina et al., 2014). Nutritional status, through amino acid-mediated protein synthesis and metabolic balance, is critical for muscle homeostasis (White, 2021). Lifestyle factors including smoking and alcohol consumption contribute to muscle damage (Xia et al., 2024; Frontera et al., 1985). Finally, chronic pathologies such as metabolic and neurological disorders, liver and kidney diseases systemically impair muscle function (Cruz-Jentoft and Sayer, 2019). In terms of testosterone, levels exhibit an age-related decline, particularly in men, while women produce significantly lower amounts (Morley et al., 1997). ​Dietary influences​ include low-fat/high-fiber diets reducing serum testosterone concentrations through hormonal modulation (Wang et al., 2005). ​Exercise has been shown to increase testosterone levels in males (Vogel et al., 1985). ​Substance use​ demonstrates divergent effects: smoking shows dose-dependent increases in testosterone (Svartberg et al., 2003), whereas alcohol dependence disrupts hepatic testosterone metabolism, paradoxically elevating levels in chronic users (Walter et al., 2007). ​Systemic pathologies​ such as diabetes mellitus and CKD impair Leydig cell function through oxidative stress and inflammatory pathways, ultimately causing hypogonadism (Leisegang et al., 2021). Several important covariates, such as genetic factors, inflammatory markers, detailed dietary data, and other hormone levels, were not included in the analysis due to ​data limitations​ in NHANES. Thus, the following covariates were included: sex, age, race, educational level, marital status, poverty-income ratio (PIR), nutrients (energy, protein, carbohydrates, fiber, and fat), physical activity, BMI, and comorbidities (hypertension, diabetes, heart failure, and stroke). Information on sociodemographic factors, lifestyle factors, and comorbidities was collected through interviews and questionnaires. Specifically, race was categorized as non-Hispanic white, non-Hispanic black, Mexican American, and other. Educational level was classified as less than high school (<9 years), high school or equivalent (9–12 years), or higher than high school (>12 years). The marital status was classified as married or cohabiting and living alone. Family income was categorized into three groups based on PIR: low income (<1.3), medium income (1.3–3.5), and high income (>3.5). Data on total energy, protein, carbohydrate, fiber, and fat intakes were collected through a 24-hour dietary recall interview. Physical activity was assessed using the Global Physical Activity Questionnaire, which covers three domains: work-, transportation-, and leisure-time-related activities. Activity levels were converted to metabolic equivalent (MET) scores, with 8.0 MET for one minute of vigorous activity and 4.0 MET for one minute of moderate or transported physical activity. Total physical activity was defined as the total number of MET hours/week (Ainsworth et al., 2011). Smokers were defined as individuals who had smoked at least 100 cigarettes in their lifetime. Participants who answered “Yes” to the question, “In any one year, have you had at least 12 drinks of any type of alcoholic beverage?“, were classified as alcohol drinkers. Comorbidities were assessed using a questionnaire that asked participants, “Have you ever been told by a doctor or other health professional that you had … “. The following pre-existing disorders were considered: hypertension, diabetes, congestive heart failure, stroke, chronic bronchitis, liver condition, arthritis, and cancer or malignancy. The estimated glomerular filtration rate (eGFR) was calculated using the Chronic Kidney Disease Epidemiology Collaboration (CKD-EPI) equation, with CKD defined as an eGFR below 60 mL/min/1.73 m^2^. This comprehensive inclusion of covariates ensured a robust analysis by accounting for multiple factors that could influence the relationship between testosterone levels and muscle mass and strength.

### 2.6 Statistical analysis

To produce nationally representative estimates, we accounted for a complex sampling design and sampling weights. Specifically, the dietary day 1 sample weight (WTDRD1) was used for the weighted analysis, with the sampling weight calculated as WTDRD1/2. Patient characteristics were stratified according to sex and divided into testosterone quartiles. Continuous variables are represented as mean and standard error of the mean (SEM) when normally distributed, or as median and interquartile range (IQR) when skewed. Categorical variables are presented as unweighted numbers and weighted percentages. Analysis of variance (ANOVA) or the Kruskal–Wallis test was used for continuous variables. Chi-square tests were used for categorical variables to determine variances between participant characteristics across the testosterone quartiles.

Serum testosterone levels were log_2_-transformed to approximate a normal distribution and divided into quartiles, with the lowest quartile serving as the reference group. Regression analyses were sex-stratified to account for biological differences in sex hormone levels between the sexes. Linear regression models were used to evaluate the associations among testosterone, ALM_BMI_ and GS_MAX_. Logistic regression models were used to examine the odds ratio (OR) and 95% confidence interval (95% CI) for the association between testosterone levels and low muscle mass and strength. In our study, we employed a multi-step approach to address potential confounding variables in the regression models. All pre-specified covariates were included simultaneously in multivariable models to adjust for known confounders and avoid stepwise selection biases. This approach minimizes residual confounding and aligns with best practices for observational studies. Model 1: Adjusted for sociodemographic variables including age, race, education level, marital status and family income (For GS_MAX_, additionally adjusted for BMI). Model 2: Further adjusted for dietary variables (energy, protein, carbohydrates, fiber, and fat) and physical activity. Model 3: Additionally adjusted for smoking status, alcohol consumption, and comorbidities (hypertension, diabetes, heart failure, stroke, chronic bronchitis, liver disease, chronic kidney disease, arthritis, and cancer). The calculation of *P* for trend was conducted by assigning median values to testosterone quartiles and testing linearity in regression models. For female participants, menopausal status was included as an additional variable in Model 3. Restricted cubic splines (RCS) were used to test for linearity and further investigate the dose-response relationship between testosterone and ALM_BMI_ in men, adjusting for confounding factors consistent with Model 3. Interaction and subgroup analyses were conducted to determine the stability of the relationship between testosterone and ALM_BMI_ across different populations stratified by age, marital status, family income, BMI, protein intake, and smoking and alcohol consumption. *P* for interaction was derived from likelihood ratio tests comparing models with and without interaction terms. Due to the parameter estimates may be biased as a result of the large proportion of missing value in covariables, we used multiple imputations based on five replications for individuals with missing data about covariates.

All data analyses and visualizations were conducted using R (version 4.3.1; The R Foundation) and Free Statistics software (version 1.9.2; Beijing Free Clinical Medical Technology Co., Ltd., Beijing, China). *P* < 0.05 was considered statistically significant.

## 3 Results

### 3.1 Characteristics of the participants

A total of 4,495 participants selected from NHANES 2011–2014 were involved in our study. The essential characteristics of excluded and included participants are presented in Supplementary Table S1. The baseline clinical characteristics of the study participants, which represent approximately 116.9 million adults in the United States are summarized in Table 1. The weighted mean age was 39.2 years (standard error [SE], 0.2), and 52.5% were men. Total testosterone levels were 20.2-fold higher in men than in women [423.8 (3.6) vs 21.0 (15.0) ng/dL; *P* < 0.001]. Individuals with higher total testosterone levels tended to be younger, living alone, alcohol consumers, with lower BMI, family income, and higher protein intake, total physical activity, and ALM_BMI_. Additionally, they had a lower prevalence of hypertension, diabetes, liver disease, arthritis, and low muscle mass.

**TABLE 1:** Characteristics of participants by sex-stratified quartiles of serum testosterone in the NHANES 2011–2014 cycles.

Characteristics		*Q*1	*Q*2	*Q*3	*Q*4	
	Total	♂ ≤298.3 ♀ ≤14.8	♂ 298.3–398.3 ♀ 14.8–21.3	♂ 398.8–518.3 ♀ 21.3–30.1	♂ ≥518.5 ♀ ≥30.2	*P*-value
No.	4,495	1,124	1,122	1,123	1,126	
Male, (%)	2,330 (52.5)	583 (53.2)	582 (51.4)	582 (52.1)	583 (53.3)	0.881
Age, years	39.2 (0.2)	43 (0.3)	40.9 (0.4)	38 (0.3)	34.7 (0.4)	<0.001
Testosterone, ng/dL	185.2 (388.9)	131.1 (235.7)	300.9 (332.7)	404.3 (428.7)	528.4 (588.1)	<0.001
Male, ng/dL	423.8 (3.6)	229.7 (2.2)	349.6 (1.2)	453.2 (1.3)	658.2 (5.4)	<0.001
Female, ng/dL	21.0 (15.0)	10.9 (4.2)	18.3 (3.1)	25.2 (4.2)	39.0 (13.5)	<0.001
Race, (%)						0.043
Non-Hispanic White	1881 (64.7)	460 (65.7)	481 (65)	449 (64.2)	491 (64.0)	
Non-Hispanic Black	943 (10.8)	231 (10.2)	201 (9.0)	252 (12.4)	259 (11.9)	
Mexican American	551 (9.6)	135 (8.6)	164 (12.0)	131 (8.0)	121 (9.7)	
Others	1,120 (14.8)	298 (15.4)	276 (14.1)	291 (15.4)	255 (14.4)	
BMI, kg/m^2^	28.6 (0.1)	29.8 (0.2)	28.9 (0.2)	28.4 (0.2)	27.1 (0.2)	<0.001
Education level, (%)						0.379
Less than high school	181 (2.8)	64 (3.9)	43 (2.7)	35 (2.1)	39 (2.5)	
High school or equivalent	1,503 (30.6)	362 (28.6)	355 (30.1)	374 (30.8)	412 (32.7)	
Above high school	2,811 (66.7)	698 (67.5)	724 (67.2)	714 (67.1)	675 (64.9)	
Marital status, (%)						<0.001
Married or living with a partner	2,596 (58.9)	724 (67.5)	703 (64.0)	645 (57.1)	524 (47.1)	
Living alone	1899 (41.1)	400 (32.5)	419 (36.0)	478 (42.9)	602 (52.9)	
Family income, (%)						0.027
PIR <1.3	1,528 (25.5)	348 (22.4)	361 (24.5)	361 (24.5)	458 (30.2)	
PIR 1.3–3.5	1,493 (33.1)	374 (32.5)	350 (31.2)	403 (36.0)	366 (33.1)	
PIR >3.5	1,474 (41.4)	402 (45.1)	411 (44.3)	359 (39.5)	302 (36.7)	
Energy intake, kcal/day	2,279.4 (15.1)	2,257.2 (28.9)	2,203.5 (27.9)	2,332.4 (31.0)	2,327.6 (32.7)	0.103
Protein intake, g/day	87.4 (0.7)	87 (1.3)	83.8 (1.2)	87.6 (1.3)	91.4 (1.5)	0.033
Carbohydrate intake, g/day	271.6 (1.9)	272.6 (3.7)	264.9 (3.6)	277.6 (3.9)	271.7 (4.2)	0.350
Fiber intake, g/day	15.4 (13.0)	15.9 (13.8)	15.6 (12.9)	15.0 (12.7)	15.1 (12.5)	0.413
Fat intake, g/day	78.1 (55.9)	75.6 (56.8)	77.2 (53.3)	83.5 (54.9)	77.2 (57.1)	0.378
Total physical activity, MET-min/week	1740.0 (4,403.1)	1,320.0 (3,360.0)	1,520.0 (3,960.0)	1920.0 (4,880.0)	2,400.0 (5,400.0)	<0.001
Smoker, (%)	1832 (42.4)	432 (40.4)	437 (41.5)	442 (43.2)	521 (44.4)	0.512
Alcohol drinker, (%)	3,509 (83.1)	835 (82.3)	872 (83)	873 (80.4)	929 (86.5)	0.027
Hypertension, (%)	1,048 (23.0)	339 (27.8)	285 (26.3)	255 (21.9)	169 (16.2)	<0.001
Diabetes, (%)	310 (5.5)	128 (8.1)	79 (5.4)	62 (4.8)	41 (3.7)	0.013
Heart failure, (%)	44 (0.9)	14 (1.4)	10 (0.9)	13 (0.9)	7 (0.5)	0.256
Stroke, (%)	55 (1.0)	19 (1.1)	10 (1.0)	17 (1.1)	9 (0.9)	0.966
Chronic bronchitis, (%)	202 (5.3)	62 (6.8)	44 (3.3)	46 (5.6)	50 (5.4)	0.076
Liver disease, (%)	126 (2.4)	41 (3.6)	24 (1.9)	36 (2.6)	25 (1.5)	0.029
CKD, (%)	38 (0.6)	13 (0.9)	11 (0.6)	7 (0.6)	7 (0.4)	0.585
Arthritis, (%)	587 (White, 2021)	190 (18.4)	171 (16.4)	131 (12.7)	95 (8.3)	<0.001
Cancer, (%)	138 (4.4)	39 (4.8)	38 (5.9)	32 (3.9)	29 (2.9)	0.232
ALM_BMI_	0.8 (0)	0.8 (0)	0.8 (0)	0.8 (0)	0.9 (0)	<0.001
Low muscle mass, (%)	325 (6.8)	119 (9.9)	93 (7.4)	71 (6.1)	42 (4.0)	0.003
GS_MAX_, kg	40.6 (0.2)	40.4 (0.4)	40.1 (0.3)	40.9 (0.3)	41.1 (0.3)	0.258
Low muscle strength, (%)	37 (1.2)	11 (1.9)	12 (1.5)	4 (0.6)	10 (0.8)	0.326

Data are presented as unweighted number (weighted percentage) for categorical variables and mean (SE) for continuous variables. *Q*, quartiles; BMI, body mass index; PIR, poverty income ratio; MET: metabolic equivalent; CKD, chronic kidney disease; ALM, appendicular lean mass; GS, grip strength.

### 3.2 Associations between testosterone levels and ALM_BMI_ and GS_MAX_

**FIGURE 2:**
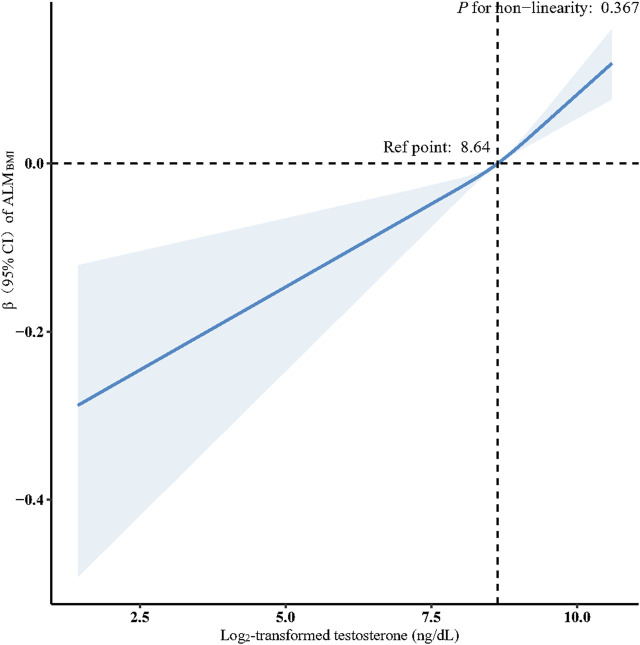
Association between log_2_-transformed testosterone and ALM_BMI_ in males. Data were fit by a multivariable linear regression model based on restricted cubic splines. Data were adjusted for age, race, educational level, marital status, family income, energy intake, protein intake, carbohydrate intake, dietary fiber, total fat intake, total physical activity, smoker, alcohol drinker, hypertension, diabetes, heart failure, stroke, chronic bronchitis, liver disease, chronic kidney disease, arthritis, and cancer (model 3). Here the median log_2_-transformed testosterone was defined as the reference standard. Solid and dashed lines indicate the predicted value and 95% CI.

Multivariate linear regression analyses of the association between serum testosterone levels and ALM_BMI_ and GS_MAX_ in male participants are shown in Table 2. A significant positive association was discovered between serum testosterone and ALM_BMI_ when analyzed as a continuous variable and subjected to log_2_-transformation in the crude model (β: 0.06, 95% CI: 0.04–0.07, *P* < 0.001). This association persisted after adjusting for sociodemographic and dietary variables, total physical activity, smoking status, alcohol consumption status, and comorbidities (Model 3). When testosterone was expressed in quartiles, participants in the highest testosterone quartile had a 0.09 increasement in ALM_BMI_ compared to those in the lowest testosterone quartile (β: 0.09, 95% CI: 0.07–0.11, *P* < 0.001). After adjusting for potential confounders, this association remained independent (β: 0.08, 95% CI: 0.04–0.11, *P* = 0.005). Furthermore, a linear relationship was observed between testosterone levels and ALM_BMI_ after adjusting for all potential confounding factors (nonlinearity: *P* = 0.367) (Figure 2). No clinically meaningful association was observed between testosterone and GS_MAX_. In women, testosterone levels were not associated with ALM_BMI_ or GS_MAX_ (Supplementary Table S2).

**TABLE 2:** Association between serum testosterone levels and ALM_BMI_ and GS_MAX_ in males.

Variable	Crude β (95% CI)	*P*-value	Model 1 β (95% CI)	*P*-value	Model 2 β (95% CI)	*P*-value	Model 3 β (95% CI)	*P*-value
ALM_BMI_								
Testosterone (log_2_)	0.06 (0.04, 0.07)	<0.001	0.05 (0.04, 0.07)	<0.001	0.05 (0.04, 0.07)	<0.001	0.05 (0.03, 0.07)	<0.001
Testosterone by quartiles								
*Q*1	0 (References)		0 (References)		0 (References)		0 (References)	
*Q*2	0.02 (−0.00, 0.04)	0.079	0.02 (−0.00, 0.04)	0.055	0.02 (−0.00, 0.05)	0.059	0.02 (−0.01, 0.06)	0.149
*Q*3	0.06 (0.04, 0.08)	<0.001	0.05 (0.03, 0.07)	<0.001	0.05 (0.03, 0.07)	<0.001	0.05 (0.02, 0.08)	0.017
*Q*4	0.09 (0.07, 0.11)	<0.001	0.08 (0.06, 0.11)	<0.001	0.08 (0.06, 0.10)	<0.001	0.08 (0.04, 0.11)	0.005
*P* for trend		<0.001		<0.001		<0.001		<0.001
GS_MAX_								
Testosterone (log_2_)	0.16 (−0.75, 1.06)	0.725	1.37 (0.29, 2.45)	0.015	1.17 (0.07, 2.27)	0.038	1.16 (−0.26, 2.58)	0.086
Testosterone by quartiles								
*Q*1	0 (References)		0 (References)		0 (References)		0 (References)	
*Q*2	−0.56 (−2.07, 0.96)	0.459	0.20 (−2.02, 0.96)	0.792	0.07 (−1.50, 1.64)	0.926	0.04 (−3.11,3.19)	0.961
*Q*3	−0.01 (−1.43, 1.41)	0.989	1.05 (−1.52, 1.28)	0.193	0.88 (−0.75, 2.51)	0.263	0.85 (−2.40, 4.10)	0.378
*Q*4	0.03 (−1.60, 1.65)	0.975	1.81 (−1.47, 1.54)	0.053	1.45 (−0.43, 3.32)	0.119	1.45 (−2.25,5.15)	0.233
*P* for trend		0.800		0.034		0.082		0.132

Crude: not adjusted. Model 1: Adjusted for age, race, educational level, marital status, and family income (For GS_MAX_, additionally adjusted for BMI). Model 2: Adjusted for model 1, additionally adjusted for energy intake, protein intake, carbohydrate intake, dietary fiber, total fat intake and total physical activity. Model 3: Adjusted for model 2, additionally adjusted for smoker, alcohol drinker, hypertension, diabetes, heart failure, stroke, chronic bronchitis, liver disease, chronic kidney disease, arthritis, and cancer. β, unstandardized coefficient; 95% CI, 95% confidence interval.

### 3.3 Associations between testosterone levels and low muscle mass and strength

Logistic regression models showed that higher testosterone levels were associated with a decreased risk of low muscle mass (OR: 0.40, 95% CI: 0.24–0.67, *P* = 0.006) in men (Table 3). This association was maintained when the testosterone levels were transformed into quartiles. Individuals in the highest testosterone quartile had an 80% lower risk of low muscle mass compared to those in the lowest testosterone quartile (OR: 0.20, 95% CI: 0.06–0.65, *P* = 0.023). In men, no correlation was observed between testosterone levels and low muscle strength. In women, testosterone levels were not associated with low muscle mass or strength (Supplementary Table S3).

**TABLE 3:** Association between testosterone levels and low muscle mass and strength in males.

Variable	Crude OR (95% CI)	*P*-value	Model 1 OR (95% CI)	*P*-value	Model 2 OR (95% CI)	*P*-value	Model 3 OR (95% CI)	*P*-value
Low muscle mass								
Testosterone (log_2_)	0.43 (0.28, 0.66)	<0.001	0.39 (0.25, 0.59)	<0.001	0.40 (0.25, 0.63)	<0.001	0.40 (0.24, 0.67)	0.006
Testosterone by quartiles								
*Q*1	1 (References)		1 (References)		1 (References)		1 (References)	
*Q*2	0.52 (0.31, 0.88)	0.015	0.48 (0.28, 0.81)	0.009	0.50 (0.29, 0.86)	0.017	0.51 (0.22, 1.15)	0.078
*Q*3	0.44 (0.25, 0.78)	0.007	0.42 (0.24, 0.73)	0.004	0.45 (0.25, 0.80)	0.010	0.45 (0.20, 1.00)	0.051
*Q*4	0.21 (0.09, 0.47)	<0.001	0.18 (0.08, 0.41)	<0.001	0.19 (0.08, 0.45)	<0.001	0.20 (0.06, 0.65)	0.023
*P* for trend		<0.001		<0.001		<0.001		0.007
Low muscle strength								
Testosterone (log_2_)	0.55 (0.35, 0.84)	0.008	0.47 (0.28, 0.77)	0.005	0.653 (0.34, 0.85)	0.011	0.51 (0.25, 1.04)	0.059
Testosterone by quartiles								
*Q*1	1 (References)		1 (References)		1 (References)		1 (References)	
*Q*2	0.77 (0.27, 2.16)	0.605	0.65 (0.23, 1.85)	0.401	0.75 (0.26, 2.20)	0.575	0.72 (0.09, 5.80)	0.563
*Q*3	0.14 (0.02, 0.88)	0.036	0.11 (0.02, 0.65)	0.017	0.15 (0.02, 0.98)	0.048	0.12 (0.00, 8.74)	0.169
*Q*4	0.28 (0.06, 1.28)	0.098	0.20 (0.04, 0.92)	0.040	0.26 (0.06, 1.19)	0.078	0.29 (0.01, 9.72)	0.270
*P* for trend		0.046		0.017		0.036		0.128

Crude: not adjusted. Model 1: Adjusted for age, race, educational level, marital status, and family income (For low muscle strength, additionally adjusted for BMI). Model 2: Adjusted for model 1, additionally adjusted for energy intake, protein intake, carbohydrate intake, dietary fiber, total fat intake and total physical activity. Model 3: Adjusted for model 2, additionally adjusted for BMI, smoker, alcohol drinker, hypertension, diabetes, heart failure, stroke, chronic bronchitis, liver disease, chronic kidney disease, arthritis, and cancer. OR, odds ratio; 95% CI, 95% confidence interval.

### 3.4 Subgroup analyses and sensitivity analysis

**FIGURE 3:**
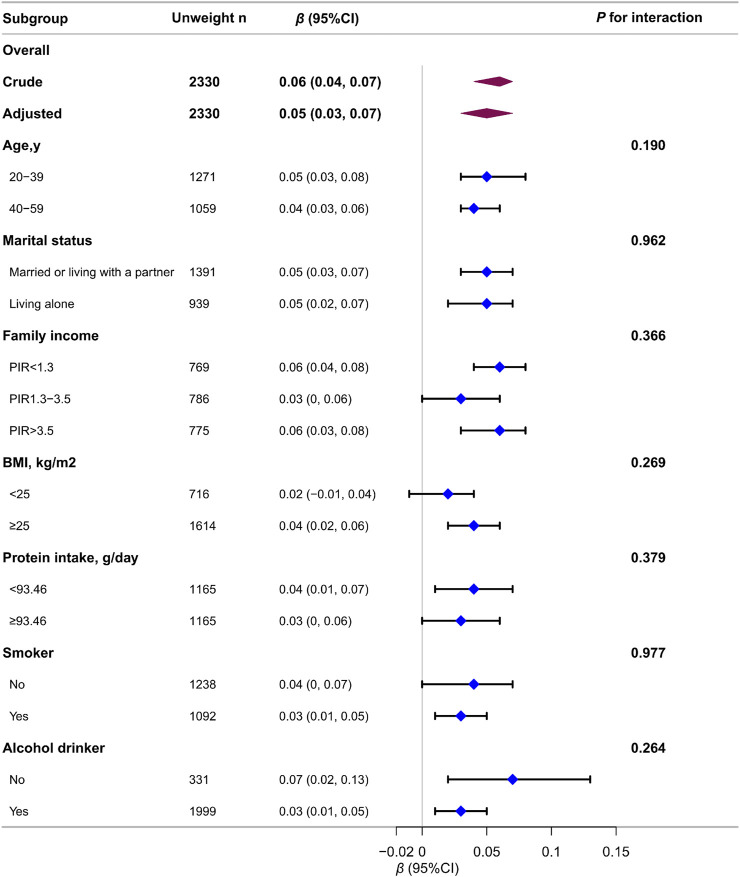
Association between log_2_-transformed testosterone levels and ALM_BMI_ in males according to the general characteristics. Each stratification was adjusted for all variables (age, race, educational level, marital status, family income, energy intake, protein intake, carbohydrate intake, dietary fiber, total fat intake, total physical activity, smoker, alcohol drinker, hypertension, diabetes, heart failure, stroke, chronic bronchitis, liver disease, chronic kidney disease, arthritis, and cancer) except the stratification factor itself.

Stratified and interaction analyses were conducted to determine whether the association between testosterone levels and ALM_BMI_ were consistent across several subgroups (Figure 3). The results showed no significant interactions in any subgroup when stratified by age, marital status, family income, BMI, protein intake, or smoking or alcohol consumption status. Of the 8,344 appropriate participants, 3,849 (46.1%) were further excluded for unavailable data. To address missing data in the analysis, multiple imputations were implemented, which yielded consistent results (Supplementary Table S4).

## 4 Discussion

Our analysis of a nationally representative cross-sectional dataset revealed that serum total testosterone was positively associated with ALM_BMI_ and inversely associated with low muscle mass in men, independent of key covariates and potential confounders, such as diet, physical activity, and comorbidities. However, no significant association was observed between testosterone levels and GS_MAX_ or low muscle strength in men. Serum testosterone levels were not associated with muscle mass or muscle strength in women.

Muscle mass and strength decline with age, with strength deterioration occurring threefold faster than mass loss (Goodpaster et al., 2006). Serum testosterone levels also decline by ∼110 ng/dL per decade in older adults (Morley et al., 1997), prompting interest in their interplay with muscle health. Existing studies have documented inconsistent associations between testosterone and muscle outcomes. For example, a recent study in premenopausal women found that free testosterone (FT) levels were associated with muscle mass, but neither FT nor total testosterone was linked to handgrip strength (Alexander et al., 2021). Another study involving 1,879 individuals aged 70–84 years showed that low FT levels predicted decreased handgrip strength in women and reduced physical performance in men, but not muscle mass loss after 2 years of follow-up (Shin et al., 2021). Intervention studies further highlight the impact of testosterone on muscle metabolism. Androgen deprivation therapy (ADT), commonly used in prostate cancer treatment, has been shown to cause significant reductions in lean mass (Chang et al., 2014) and muscle cross-sectional areas (Storer et al., 2017). Conversely, testosterone replacement therapy (TRT) has been found to improve muscle mass and strength in hypogonadal men. A recent double-blind randomized controlled trial (RCT) reported modest but significant improvements in muscle mass and strength after 3 years of testosterone administration in older men with low-to-normal testosterone levels (Hildreth et al., 2013). However, other studies have not observed a direct effect of TRT on muscle mass or strength (Kolind et al., 2022; Barnouin et al., 2021). These inconsistencies may be due to variations in study populations, doses, modes of administration, and other factors such as diet and physical activity levels. Unlike the aforementioned studies, our research specifically focuses on young to middle-aged adults and systematically dissecting sex-based disparities in testosterone’s role in muscle health.

In men, a positive linear relationship was found between testosterone and muscle mass but not muscle strength, suggesting a disconnect between muscle mass preservation and functional strength gains. This disconnect may arise from multifaceted biological and physiological mechanisms. First, muscle strength depends not only on mass but also on neuromuscular efficiency and motor unit recruitment (Sale, 1988). Aging diminishes the number and function of alpha motor neurons, leading to fewer muscle fibers being activated during contraction. Even if muscle mass is preserved, impaired neural drive reduces force generation (Delmonico et al., 2009). Second, type II fibers, critical for strength and power, exhibit more pronounced atrophy and decrease in number with age, while type I fibers are less affected (Lexell, 1995). Third, age-related mitochondrial dysfunction reduces ATP synthesis, limiting muscle contractile capacity. This affects strength disproportionately to mass (Short et al., 2005). Lastly, aging blunts the muscle protein synthesis response to anabolic stimuli (e.g., testosterone, insulin, exercise), impairing strength maintenance (Mitchell et al., 2012). In addition, testosterone alone may not be a key determinant of strength when considering the broader context of health and lifestyle. Diet is a key factor influencing testosterone levels and muscle health. A Western-style diet, characterized by high consumption of bread, pastries, dairy products, and desserts, is associated with lower testosterone levels and an increased risk of hypogonadism (Hu et al., 2018). Conversely, adherence to a Mediterranean diet may improve testosterone levels (La et al., 2018). Physical activity is another critical factor. Regular exercise, especially resistance training, has been shown to potentiate muscle hypertrophy and increase testosterone levels (Vingren et al., 2010). In contrast, a sedentary lifestyle is associated with lower testosterone levels and musculoskeletal diseases (Park et al., 2020). Smoking and drinking behavior not only affects testosterone levels, but also negatively impacts muscle health (Xia et al., 2024; Ko et al., 2020). Health conditions like diabetes, chronic kidney disease, and heart failure can directly impact muscle strength due to their effects on overall health and metabolic processes while neurological conditions affecting the nervous system can also influence muscle strength by altering muscle control and function (Cruz-Jentoft and Sayer, 2019).

In women, no correlation was observed between testosterone levels and ALM_BMI_, low muscle mass, GS_MAX_, or low muscle strength. The sex-specific differences in the relationship between testosterone levels and muscle health may be attributed to several biological and hormonal factors. First, women in our cohort had testosterone levels approximately 20-fold lower than men, which may be below the threshold required to exert anabolic effects on muscle tissue (Shin et al., 2021). Alternatively, the effect of testosterone on muscle health in women may be less significant than that of the other factors including age, nutrition, estrogen, physical activity, lifestyle and health status. Second, variations in androgen receptor expression and sensitivity between men and women may influence how testosterone affects muscle tissue. Studies have shown that androgen receptor density and activity can differ between sexes, potentially explaining the disparate effects of testosterone on muscle health (Schuppe et al., 2017). Third, men and women have different muscle fiber compositions, with men generally having a higher proportion of type II (fast-twitch) fibers (Staron et al., 2000), which are more responsive to testosterone and contribute to greater muscle mass and strength. Nationally representative data revealed significant sex-based variations in testosterone levels: ​in men, the 10th-90th percentile range for total testosterone was 150–698 ng/dL, while ​in women​ it spanned 7.1–49.8 ng/dL (Vesper et al., 2015). Furthermore, physiological testosterone levels exhibited a positive correlation with lean mass and an inverse association with fat mass in males (Mouser et al., 2016). ​Conversely, in pre-menopausal women, total testosterone levels demonstrated ​no significant relationship​ with either lean mass or handgrip strength (Alexander et al., 2021).

While our study found no significant association between testosterone levels and grip strength, the distinction between muscle *strength* and *power* is critical as grip strength measurements in NHANES may not fully capture dynamic power-related tasks. Muscle power, which reflects the ability to generate force rapidly, declines earlier and more precipitously with aging than muscle strength and is more strongly associated with functional limitations and fall risk in older adults (Bean et al., 2003). Studies have demonstrated that testosterone administration improves stair-climbing power, leg press power, and chest press strength and power (Storer et al., 2017). These effects are dose-dependent, with higher testosterone levels correlating with greater improvements in muscle performance (Storer et al., 2003). While testosterone supplementation improves maximal voluntary strength and leg power, its effects on endurance performance and overall physical function in older adults remain unclear (Bhasin et al., 2001). Future studies incorporating validated measures of muscle power would provide a more comprehensive understanding of testosterone’s role in muscle function and its clinical implications.

The large sample size of the NHANES dataset minimized non-sampling and measurement errors due to the rigorous study design and data-processing criteria. We accounted for complex sampling weights and sample designs to ensure the representativeness and generalizability of our findings. However, this study has several limitations. This study focused on young to middle-aged adults (aged 20–59 years) in the United States, as the NHANES dataset does not include individuals outside this age range in the DXA scans examination. While our findings provide valuable insights into the relationship between testosterone levels and muscle health in this demographic, the generalizability to older adults (≥60 years) or non-U.S. populations remain uncertain. Future studies incorporating broader age ranges and diverse cohorts are needed to validate these findings and explore potential age-related differences. The cross-sectional nature of our study is another limitation, as it precludes the establishment of causality. Our findings are based on a snapshot of data collected at a single point in time, which means that we cannot infer temporal relationships between testosterone levels and muscle health outcomes. Longitudinal studies are needed to determine whether the observed associations are causal and to explore the potential bidirectional relationships between testosterone levels and muscle mass/strength over time. Despite accounting for various lifestyle- and health-related confounders, residual confounding may still exist. For example, certain genetic variants may affect muscle fiber type distribution, muscle protein synthesis, and androgen receptor sensitivity (Ahmetov et al., 2012). Chronic low-grade inflammation is common in older adults and can affect muscle health independently of testosterone levels (Degens, 2010). Detailed dietary intake data, such as specific micronutrient levels (e.g., vitamin D, calcium), were not fully accounted for in our analysis (Annweiler et al., 2009). Other hormones, such as estrogen, growth hormone, and insulin-like growth factor-1 (IGF-1), interact with testosterone and could influence muscle health. Self-reported data on diet, physical activity, and comorbidities are susceptible to recall bias. Moreover, different types of physical activity—such as aerobic and resistance exercise—may have varying impacts on muscle health. However, the NHANES dataset lacks detailed information on the specific types of physical activity, limiting our ability to draw more nuanced conclusions. Additionally, free testosterone is generally considered a more accurate indicator of biologically active hormone levels compared to total testosterone. Unfortunately, our analysis was constrained by the available data, as the NHANES dataset does not provide measurements of free testosterone. Finally, given the differences in baseline data between included and excluded participants, it is imprudent to dismiss the potential impact of non-random omissions on our results.

## 5 Conclusion

Our findings suggest an epidemiological association between serum testosterone levels and muscle mass, but not muscle strength, in young to middle-aged males. Additional research is necessary to explore the complexities of this relationship further and to address gaps observed in female participants.

## Data Availability

The original contributions presented in the study are included in the article/Supplementary Material, further inquiries can be directed to the corresponding author/s.
