# Supplementary material for: Testosterone levels positively linked to muscle mass but not strength in adult males aged 20–59 years: a cross-sectional study
Source: Front Physiol. 2025 Apr 15;16:1512268. doi: 10.3389/fphys.2025.1512268 (PMC12037588; doi:10.3389/fphys.2025.1512268)
Supplement: Supplementary file 1 [file Table1.docx]

**Supplementary Table S1** Characteristics of included and excluded participants.

|  | Include (4495) | Exclude (3849) | *P*-value |
| --- | --- | --- | --- |
| Gender, n (%) |  |  | 0.255 |
| Male | 2330 (51.8) | 1947 (50.6) |  |
| Female | 2165 (48.2) | 1902 (49.4) |  |
| Age, years | 38.5 (11.5) | 18.0 (11.8) | <0.001 |
| Race, (%) |  |  | <0.001 |
| Non-Hispanic White | 1881 (41.8) | 990 (25.7) |  |
| Non-Hispanic Black | 943 (21.0) | 994 (25.8) |  |
| Mexican American | 551 (12.3) | 825 (21.4) |  |
| Others | 1120 (24.9) | 1040 (27.0) |  |
| Education level, (%) |  |  | <0.001 |
| Less than high school | 181 (4.0) | 73 (11.0) |  |
| High school or equivalent | 1503 (33.4) | 248 (37.2) |  |
| Above high school | 2811 (62.5) | 345 (51.8) |  |
| Marital status, (%) |  |  | 0.095 |
| Married or living with a partner | 2596 (57.8) | 408 (61.2) |  |
| Living alone | 1899 (42.2) | 259 (38.8) |  |
| Family income, (%) |  |  | <0.001 |
| PIR <1.3 | 1528 (34.0) | 1528 (46.5) |  |
| PIR 1.3–3.5 | 1493 (33.2) | 1072 (32.6) |  |
| PIR >3.5 | 1474 (32.8) | 687 (20.9) |  |
| BMI, kg/m^2^ | 28.7 (6.7) | 23.5 (6.5) | <0.001 |
| Protein intake, g/day | 87.5 (45.8) | 76.5 (43.3) | <0.001 |
| Total physical activity, MET-min/week | 1680.0 (3360.0) | 1040.0 (3240.0) | <0.001 |
| Smoker, (%) | 1832 (40.8) | 299 (32.4) | <0.001 |
| Alcohol drinker, (%) | 3509 (78.1) | 515 (57.9) | <0.001 |
| Hypertension, (%) | 1048 (23.3) | 206 (12.6) | <0.001 |
| Diabetes, (%) | 310 (6.9) | 57 (1.5) | <0.001 |
| Stroke, (%) | 55 (1.2) | 6 (0.9) | 0.472 |
| Cancer, (%) | 138 (3.1) | 16 (2.4) | 0.342 |
| Testosterone, ng/dL | 176.9 (384.7) | 26.1 (267.8) | <0.001 |
| ALM_BMI_ | 0.8 (0.2) | 0.8 (0.2) | <0.001 |
| Low muscle mass, (%) | 325 (7.2) | 994 (25.8) | <0.001 |
| GS_MAX_, kg | 40.2 (11.3) | 29.5 (11.6) | <0.001 |
| Low muscle strength, (%) | 37 (0.8) | 1109 (28.8) | <0.001 |

Abbreviation: OR, odds ratio; CI, confidence interval; BMI, body mass index; PIR, poverty income ratio; MET: metabolic equivalent; ALM, appendicular lean mass; GS, grip strength.

**Supplementary Table S2** Association between serum testosterone levels and ALM_BMI_ and GS_MAX_ in females.

| Variable | Crude  β (95% CI) | *P*-value | Model 1  β (95% CI) | *P*-value | Model 2  β (95% CI) | *P*-value | Model 3  β (95% CI) | *P*-value |
| --- | --- | --- | --- | --- | --- | --- | --- | --- |
| **ALM_BMI_** |  |  |  |  |  |  |  |  |
| Testosterone (log_2_) | 0.01 (-0.00, 0.02) | 0.080 | 0.00 (-0.01, 0.01) | 0.423 | -0.01 (-0.02, 0.00) | 0.277 | -0.01 (-0.02, 0.01) | 0.294 |
| Testosterone by quartiles |  |  |  |  |  |  |  |  |
| *Q*1 | 0 (Reference) |  | 0 (Reference) |  | 0 (Reference) |  | 0 (Reference) |  |
| *Q*2 | -0.01 (-0.03, 0.01) | 0.187 | -0.02 (-0.04, -0.00) | 0.038 | -0.02 (-0.04, -0.00) | 0.034 | -0.02 (-0.05, 0.02) |  |
| *Q*3 | 0.00 (-0.02, 0.02) | 0.882 | -0.01 (-0.03, 0.00) | 0.109 | -0.02 (-0.03, 0.00) | 0.076 | -0.02 (-0.05, 0.02) |  |
| *Q*4 | 0.01 (-0.01, 0.03) | 0.386 | -0.02 (-0.04, 0.01) | 0.127 | -0.02 (-0.05, 0.00) | 0.071 | -0.02 (-0.07, 0.02) |  |
| *P* for trend |  | 0.185 |  | 0.165 |  | 0.090 |  | 0.139 |
| **GS_MAX_** |  |  |  |  |  |  |  |  |
| Testosterone (log_2_) | 0.79 (0.42, 1.16) | <0.001 | 0.32 (-0.06, 0.71) | 0.092 | 0.25 (-0.15, 0.65) | 0.198 | 0.14 (-0.45, 0.73) | 0.508 |
| Testosterone by quartiles |  |  |  |  |  |  |  |  |
| *Q*1 | 0 (Reference) |  | 0 (Reference) |  | 0 (Reference) |  | 0 (Reference) |  |
| *Q*2 | 0.53 (-0.59, 1.66) | 0.341 | 0.34 (-0.74, 1.42) | 0.514 | 0.39 (-0.69, 1.47) | 0.451 | 0.08 (-0.51, 0.67) | 0.886 |
| *Q*3 | 1.44 (0.35, 2.52) | 0.011 | 0.99 (-0.11, 2.09) | 0.075 | 1.00 (-0.15, 2.15) | 0.082 | 0.48 (-0.13, 1.09) | 0.540 |
| *Q*4 | 1.45 (0.49, 2.41) | 0.004 | 0.78 (-0.24, 1.80) | 0.127 | 0.63 (-0.43, 1.69) | 0.223 | 0.01 (-0.63, 0.65) | 0.991 |
| *P* for trend |  | <0.001 |  | 0.061 |  | 0.129 |  | 0.801 |

Crude: not adjusted. Model 1: Adjusted for age, race, educational level, marital status, and family income (For GS_MAX_, additionally adjusted for BMI). Model 2: Adjusted for model 1, additionally adjusted for energy intake, protein intake, carbohydrate intake, dietary fiber, total fat intake and total physical activity. Model 3: Adjusted for model 2, additionally adjusted for smoker, alcohol drinker, hypertension, diabetes, heart failure, stroke, chronic bronchitis, liver disease, chronic kidney disease, arthritis, and cancer. β, unstandardized coefficient; 95% CI, 95% confidence interval.

**Supplementary Table S3** Association between testosterone levels and low muscle mass and strength in females.

| Variable | Crude  OR (95% CI) | *P*-value | Model 1  OR (95% CI) | *P*-value | Model 2  OR (95% CI) | *P*-value | Model 3  OR (95% CI) | *P*-value |
| --- | --- | --- | --- | --- | --- | --- | --- | --- |
| **Low muscle mass** |  |  |  |  |  |  |  |  |
| Testosterone (log_2_) | 0.87 (0.66, 1.13) | 0.278 | 1.05 (0.76, 1.45) | 0.756 | 1.07 (0.77, 1.51) | 0.657 | 1.11 (0.70, 1.76) | 0.576 |
| Testosterone by quartiles |  |  |  |  |  |  |  |  |
| *Q*1 | 1 (Reference) |  | 1 (Reference) |  | 1 (Reference) |  | 1 (Reference) |  |
| *Q*2 | 1.24 (0.62, 2.47) | 0.528 | 1.36 (0.65, 2.87) | 0.398 | 1.34 (0.63, 2.88) | 0.420 | 1.45 (0.29, 7.28) | 0.424 |
| *Q*3 | 0.97 (0.51, 1.84) | 0.925 | 1.34 (0.63, 2.86) | 0.433 | 1.35 (0.62, 2.96) | 0.419 | 1.35 (0.28, 6.75) | 0.477 |
| *Q*4 | 0.84 (0.38, 1.83) | 0.644 | 1.30 (0.48, 3.53) | 0.592 | 1.35 (0.48, 3.77) | 0.539 | 1.45 (0.18, 11.77) | 0.528 |
| *P* for trend |  | 0.432 |  | 0.566 |  | 0.506 |  | 0.487 |
| **Low muscle strength** |  |  |  |  |  |  |  |  |
| Testosterone (log_2_) | 1.13 (0.46, 2.78) | 0.786 | 1.49 (0.69, 3.25) | 0.297 | 1.80 (0.60, 5.42) | 0.273 | 1.77 (0.23, 12.52) | 0.438 |
| Testosterone by quartiles |  |  |  |  |  |  |  |  |
| *Q*1 | 1 (Reference) |  | 1 (Reference) |  | 1 (Reference) |  | 1 (Reference) |  |
| *Q*2 | 1.26 (0.17, 9.27) | 0.811 | 1.57 (0.20, 12.27) | 0.654 | 1.46 (0.14, 14.91) | 0.732 | 1.39 (0.00, 17.80) | 0.865 |
| *Q*3 | 2.52 (0.20, 31.37) | 0.459 | 3.20 (0.34, 29.88) | 0.291 | 4.39 (0.45, 43.34) | 0.187 | 5.86 (0.00, 68.86) | 0.330 |
| *Q*4 | 2.08 (0.31, 13.91) | 0.437 | 5.06 (0.78, 33.03) | 0.086 | 7.10 (0.73, 69.27) | 0.086 | 6.50 (0.00, 95.53) | 0.362 |
| *P* for trend |  | 0.318 |  | 0.05 |  | 0.044 |  | 0.142 |

Crude: not adjusted. Model 1: Adjusted for age, race, educational level, marital status, and family income (For low muscle strength, additionally adjusted for BMI). Model 2: Adjusted for model 1, additionally adjusted for energy intake, protein intake, carbohydrate intake, dietary fiber, total fat intake and total physical activity. Model 3: Adjusted for model 2, additionally adjusted for BMI, smoker, alcohol drinker, hypertension, diabetes, heart failure, stroke, chronic bronchitis, liver disease, chronic kidney disease, arthritis, and cancer. OR, odds ratio; 95% CI, 95% confidence interval.

**Supplementary Table S4** Association between serum testosterone levels and ALM_BMI_ and low muscle mass in males using complete case analysis and multiply imputed data.

|  | ^*^Complete cases | | ^*^Number with missing data | |
| --- | --- | --- | --- | --- |
|  | OR (95% CI) | *P*-value | OR (95% CI) | *P*-value |
| ALM_BMI_ |  |  |  |  |
| Testosterone (log_2_) | 0.05 (0.03, 0.07) | <0.001 | 0.06 (0.05, 0.06) | <0.001 |
| Low muscle mass |  |  |  |  |
| Testosterone (log_2_) | 0.40 (0.24, 0.67) | 0.006 | 0.36 (0.31, 0.42) | <0.001 |

^*^Adjusted covariates: age, race, educational level, marital status, family income, energy intake, protein intake, carbohydrate intake, dietary fiber, total fat intake, total physical activity, smoker, alcohol drinker, hypertension, diabetes, heart failure, stroke, chronic bronchitis, liver disease, chronic kidney disease, arthritis, and cancer.

The results were generally robust when using a multiple imputation to address missing data.
